# A prospective comparison between multidisciplinary healthcare providers' clinical examination and a validated pain scale

**DOI:** 10.3389/fpain.2022.960216

**Published:** 2022-08-12

**Authors:** Rodrigo C. Menezes, Raissa L. O. Silva, María B. Arriaga, Isabella B. B. Ferreira, Thomas A. Carmo, Victor R. da Silva, Matheus L. Otero, André L. N. Gobatto, Sydney Agareno, Nivaldo M. Filgueiras Filho, Kevan M. Akrami, Bruno B. Andrade

**Affiliations:** ^1^Universidade Federal da Bahia, Faculdade de Medicina da Bahia, Salvador, Brazil; ^2^Instituto Gonçalo Moniz, Fundação Oswaldo Cruz, Salvador, Bahia, Brazil; ^3^Multinational Organization Network Sponsoring Translational and Epidemiological Research (MONSTER) Initiative, Salvador, Bahia, Brazil; ^4^Universidade Salvador (UNIFACS), Faculdade de Medicina, Salvador, Bahia, Brazil; ^5^Universidade Do Estado da Bahia (UNEB), Departamento de Ciências da Vida, Salvador, Bahia, Brazil; ^6^Intensive Care Unit, Hospital de Cidade, Salvador, Bahia, Brazil; ^7^Division of Infectious Diseases and Pulmonary Critical Care and Sleep Medicine, Department of Medicine, University of California, San Diego, San Diego, CA, United States; ^8^Escola Bahiana de Medicina e Saúde Pública (EBMSP), Salvador, Bahia, Brazil

**Keywords:** pain, critical care, pain management, Critical-Care Pain Observation Tool, pain examination

## Abstract

**Introduction:**

Unrecognized pain in the Intensive Care Unit (ICU), due to inadequate assessment and therapeutic management, is associated with increased morbidity and mortality. Despite the availability of validated pain monitoring tools, such as the Critical-Care Pain Observational Tool (CPOT), these scales are not commonly used in clinical practice, with healthcare professionals often relying on their clinical impression. Our study aims to determine the agreement between the pain examination performed by ICU professionals and the CPOT.

**Methods:**

Prospective cohort study that included critically ill patients and physicians, nurses and physiotherapists from an ICU in Bahia, Brazil. During bedside clinical rounds, the CPOT score was applied to assess the pain of hospitalized patients, and health professionals were interviewed to ascertain their perception of the patient's pain for a maximum of five consecutive days. Correlations were assessed using the Spearman rank tests. Hierarchical cluster analysis was employed to show the results of CPOT score and pain assessment by healthcare professionals at each study time. And the Kappa statistic was calculated to assess the agreement between the CPOT score vs. the pain assessment by healthcare providers.

**Results:**

One hundred one patients were included in the study with median age of 74 years (IQR 61.5–83.5), a predominance of women (55.4%) and a median SAPS 3 score of 45 (IQR 39.5–53.0). The correlation between the professional's pain assessment and the CPOT were mostly statistically significant, ranged from negligible to weak, being the highest index obtained in the evaluation of nurses on day 5 (Kappa index = 0.43, *p* = 0.005). Physician assessments were significant only in day 1. On the presence of pain, the professionals' assessments and CPOT revealed mild to a moderate agreement.

**Conclusion:**

Healthcare professional's pain assessment displayed a weak positive correlation with a validated pain scale and poor agreement amongst members of the ICU team, particularly when the pain was felt to be absent. Thus, this study highlights the importance of routine tools for pain assessment in the ICU for all members of multidisciplinary teams.

## Introduction

Pain is perhaps the most stressful event that patients experience during their stay in the intensive care unit (ICU). It is associated with an acute response to stress, characterized by changes in heart and respiratory rate, blood pressure and neuroendocrine secretion (1, 2). Appropriate assessment and treatment of pain in the ICU is critical, as it is associated with a better clinical outcomes (3). On the other hand, inadequate pain control significantly favors the post-intensive care syndrome, particularly related to common painful procedures, such as arterial line insertion or airway suctioning (4, 5).

While prior studies have highlighted the importance of pain in the ICU, many factors hinder caregiver's ability to communicate effectively with patients, including the use of sedative agents, mechanical ventilation, and changes in sensorium (6). In the absence of the patient's communication, observable behavioral and physiologic indicators become important proxies for pain assessment. Despite this, health professionals often rely on imprecise bedside assessments leading to excessive or insufficient sedation and analgesia (7).

Given the severity of life-threatening conditions in the critically ill, prompt recognition and management of pain may be overlooked. While there are validated and recommended pain assessment scales such as the Critical-Care Pain Observational Tool (CPOT) for critically ill patients, prior work demonstrates that these are rarely used routinely in the ICU (8). Recent studies indicate that the use of these scales improves care and therapeutic interventions in ICUs through the introduction of effective pain management protocols, reduction in the use of sedatives and reduced time of mechanical ventilation, thus benefiting the quality of care in these patients.

This study aims to assess the concordance between pain assessment performed by ICU professionals and a validated pain assessment scale in critically ill patients.

## Materials and methods

### Ethics approval

This research was approved by the Research Ethics Committee of the Federal University of Bahia under the number 2.249.687 and CAAE 73835317.5.0000.5577. And was conducted in compliance with the Helsinki declaration.

### Design, site, and sample

Prospective observational and analytical cohort study conducted from September to October 2017 in a tertiary ICU in Salvador, Bahia, Brazil. All patients ≥18 years old, consecutively admitted to the ICU, who provided informed consent, were included in the study. The multidisciplinary team included 13 physicians, 18 nurses, and 16 physiotherapists who also provided informed consent. Patients with a stay of fewer than 24 h, conditions that compromised the expression of pain behaviors, such as decortication or decerebration, and use of neuromuscular blockers, were excluded.

### Procedures

The demographic characteristics, clinical and laboratory data were obtained from the electronic medical records of the patients. These included: age, primary ICU admission diagnosis, comorbidities, vital signs, level of consciousness through the Glasgow Coma Scale (GCS) and the Richmond Agitation Sedation Scale (RASS) score, arterial blood gas test, hemogram, serum creatinine, total serum bilirubin, use of vasoactive drugs, analgesics, sedatives, or drugs with the potential to interfere in the patient's perception of pain, such as anxiolytics and anti-depressants. The total number and type of painful procedures performed were recorded every 24 h, including orotracheal suctioning, arterial puncture, wound care, thoracocentesis, and chest tube removal.

All researchers received the same rigorous training to properly apply the CPOT, in which each one applied the score to hospitalized patients and compared the responses to ensure that there was inter-observer agreement in order to reduce measurement bias. The behavioral parameters of CPOT included facial expression, body movements, body tension and ventilator compliance in intubated patients or vocalization in patients with spontaneous breathing. Each behavior was rated from 0 to 2, resulting in a possible total score ranging from 0 to 8, where >2 points represents the presence of pain (1). A CPOT SCORE of 3 or more was considered an unacceptable pain level, that is, pain that the healthcare team should consider additional or alternative analgesia and sedation.

In the studied ICU, daily clinical rounds are performed at the patient's bedside with the physician, physiotherapist and nurse responsible for the patient. Immediately before the visit, the researchers applied the CPOT to the patient. The study team also noted the presence or absence of tracheostomy, mechanical ventilation and ventilatory support. Then, after each patient visit, healthcare professionals were interviewed individually, being asked whether the patient was in pain and, if so, to rate the pain intensity from 0 to 10. There was no communication among the professionals regarding their pain evaluation. Patients and healthcare providers underwent daily evaluations by for up to 5 days.

### Statistical analyses

Categorical data were presented as proportions and continuous data as medians and interquartile ranges (IQR). Correlations were assessed using the Spearman rank tests. Hierarchical cluster analysis (Ward's method) was employed to show the results of CPOT score and pain assessment by nurses, physicians and physiotherapists at each study time. A *p* < 0.05 was considered statistically significant. Statistical analyses were performed using SPSS 24.0 (IBM statistics), Graphpad Prism 7.0 (GraphPad Software, San Diego, CA) and JMP 12.0 (SAS, Cary, NC, USA). Finally, the Kappa (K) statistic was calculated to assess the agreement between the presence of an unacceptable amount pain according to CPOT score vs. the pain assessment by nurses, physicians, and physiotherapists at each study time. The Kappa statistic was interpreted by Landis and Koch criteria. Bland-Altman analyses were conducted to provide a visual representation of the concordance between the methods.

## Results

**Figure 1:**
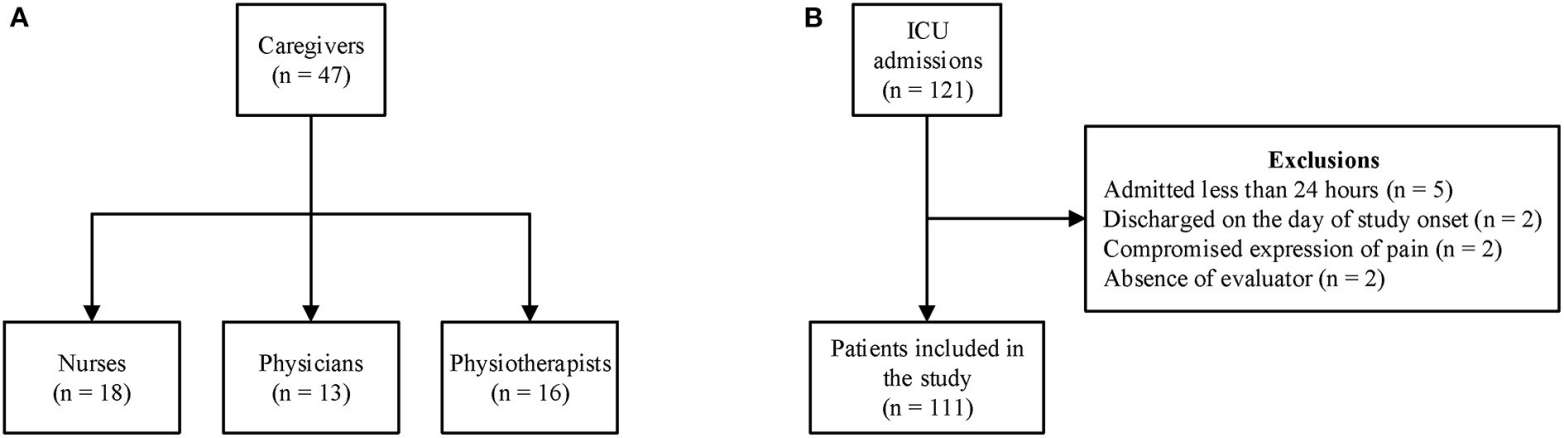
Flowchart of the study. **(A)** Distribution of healthcare professionals and **(B)** patient enrollment.

There were 111 admissions over the study period and 10 exclusions (5 had a length of stay of <24 h, two discharged on the day of study start, two for conditions that compromised the expression of pain behaviors, and 1 for absence of a member of the multidisciplinary team during the collection period). Patients were followed up for a maximum of five consecutive days, with 336 pain assessments being performed (CPOT and the interview of professionals) (Figure 1). The median age was 74 years (IQR 61.5–83.5) with a predominance of women 56 (55.4%) and the median length of stay in the ICU and in the hospital was 4 days (2.0–8.0) and 7 days (4.0–17.50), respectively. The median SAPS 3 score was 45 (39.5–53.0). There were 17 (16.8%) hospital deaths, of which 14 (13.9%) occurred in the ICU. Ninety (89.1%) of the patients included in the study were functionally independent, whereas 3 (3%) required assistance and 8 (7.9%) were restricted/bedridden at the time of admission. Table 1 highlights additional demographic characteristics of the patients.

**Table 1:** Population clinical characteristics.

**Characteristics**	**Values**
Age (years)—median (IQR)	74 (61.5–83.5)
Female sex—no. (%)	56 (55.4)
SAPS 3—median (IQR)	45 (39.5–53.0)
Hospital length of stay—median (IQR)	7 (4.0–17.5)
ICU length of stay—median (IQR)	4 (2.0–8.0)
Hospital mortality—no. (%)	17 (16.8)
**Admission type—no. (%)**
Medical	90 (89.1)
Cardiovascular	26 (25.7)
Neurologic	11 (10.9)
Respiratory	6 (5.9)
Gastrointestinal	8 (7.9)
Infectious	22 (21.8)
Surgical	11 (10.9)
Others	28 (27.7)
**Comorbidities—no. (%)**
Hypertension	74 (73.3)
Diabetes mellitus	37 (36.6)
Prior stroke	12 (11.9)
Dyslipidemia	14 (13.9)
Chronic kidney disease	15 (14.9)
Heart failure	12 (11.9)
Malignant neoplasm	7 (6.9)
**Functional status—no. (%)**
Independent	90 (89.1)
Need for assistance	3 (3)
Restricted/bedridden	8 (7.9)
Admission GCS—median (IQR)	15 (15–15)
Admission RASS—median (IQR)	0 (0–0)

Data presented as frequency n (%) or median and interquartile range (IQR). ICU, Intensive care unit; SAPS 3, Simplified acute physiology score III; GCS, Glasgow coma scale; RASS, Richmond agitation-sedation scale.

During the 5 days of evaluation, 7 patients (6.9%) required mechanical ventilation on day 1, 5 (6.1%) on day 2, 5 (7.8%) on day 3, 4 (8.5%) on day 4, and 4 (10.3%) on day 5. Analgesic use varied throughout hospitalization with 37 (36.6%) on the first day of evaluation, which increased until the third day of hospitalization. Opiate use on day 1 included 19 (18.8%) patients, though this similarly increased until the third day of evaluation. Sedatives were used in a minority of patients, the greatest use being on day 2 with 6 (7.3%) patients. The median RASS ranged from −1 to 0 on most study days. Arterial puncture was the most frequent procedure in patients (29.7, 24.4, 20.3, 17, 15.4%, on days 1, 2, 3, 4, and 5, respectively). In contrast, wound debridement was the least performed procedure (0, 1.2, 0, 2.1, 0, on days 1, 2, 3, 4, and 5, respectively). These findings are detailed in Table 2.

**Table 2:** Pain evaluation and procedures at each day.

**Characteristics**	**Day 1** **(*n* = 101)**	**Day 2** **(*n* = 82)**	**Day 3** **(*n* = 64)**	**Day 4** **(*n* = 47)**	**Day 5** **(*n* = 39)**
Mechanical ventilation use—no. (%)	7 (6.9)	5 (6.1)	5 (7.8)	4 (8.5)	4 (10.3)
Orotracheal suctioning—no. (%)	5 (0.05)	5 (0.06)	5 (7.8)	1 (2.1)	1 (2.6)
Arterial puncture—no. (%)	30 (29.7)	20 (24.4)	13 (20.3)	8 (17)	6 (15.4)
Wound debridement—no. (%)	0 (0)	1 (1.2)	0 (0)	1 (2.1)	0 (0)
Removal of chest drain—no. (%)	1 (1)	4 (4.9)	3 (4.7)	0 (0)	1 (2.6)
Wound dressing —no. (%)	12 (11.9)	14 (13.9)	15 (23.4)	9 (19.1)	7 (17.9)
Analgesic use—no. (%)	37 (36.6)	32 (31.8)	25 (39.1)	15 (14.9)	9 (23)
Opiate use—no. (%)	19 (18.8)	14 (17.1)	11 (17.2)	6 (11)	3 (7)
Sedative use—no. (%)	5 (5)	6 (7.3)	2 (3.1)	3 (6.4)	2 (5.1)
RASS—no. (%)	0	0	0	0*	0*
Total CPOT—median (IQR)	0 (0–1)	0 (0–1)	0 (0–1)	0 (0–1)	0 (0–1)
Physician assessment—median (IQR)	0 (0–3)	0 (0–1.25)	0 (0–0)	0 (0–2)	0 (0–0)
Nurse assessment—median (IQR)	0 (0–3)	0 (0–0)	0 (0–3)	0 (0–2)	0 (0–0)
Physiotherapist assessment—median (IQR)	0 (0–1)	0 (0–0)	0 (0–3)	0 (0–3)	0 (0–3)

Data presented as frequency n (%) or median and interquartile range (IQR).

^*^IQR −1–0. RASS, Richmond agitation-sedation scale; CPOT, Critical care pain observation tool.

Multidisciplinary team members included 13 (27.7%) physicians, 18 (38.3%) nurses, and 16 (34%) respiratory therapists. Professionals' pain assessments correlated insignificantly with CPOT, ranging from negligible (14% of the evaluations) to weak (86% of the evaluations). Physician evaluations demonstrated poor correlation with CPOT, only achieving significant correlation on day 1 (*r* = 0.34, *p* < 0.001). Physiotherapists' evaluations suffered less daily variation of correlation with CPOT compared with nurses and physicians, though similarly did not reach statistical significance (Table 3). There is a high range in Bland-Altman analysis with a regression line with a negative trend (Supplementary Figure 1).

**Table 3:** Correlation between CPOT pain evaluation and procedures at each day.

**Correlation coefficient CPOT vs**.	**Day 1**	**Day 2**	**Day 3**	**Day 4**	**Day 5**
Physician	0.34*	0.32	0.14	0.34	0.36
Nurse	0.34	0.3	0.32	0.16	0.42
Respiratory therapist	0.27	0.28	0.38	0.35	0.28

Correlations were assessed using the Spearman rank tests.

^*^p < 0.001.

CPOT, Critical care pain observation tool.

In the presence of pain, the overall agreement between professionals' assessments and CPOT was greater on the first day (Kappa Index = 0.36, 95% CI: 0.28–0.43; *p* < 0.001). The proportion of agreement in the presence or absence of pain varied daily between 67.1 and 76.9%, with the lowest kappa index of agreement observed in the evaluation of physicians on day 3 (Kappa Index = 0.13, *p* = 0.18) and the highest index obtained in the evaluation of nurses on day 5 (Kappa index = 0.43, *p* = 0.005).

**Figure 2:**
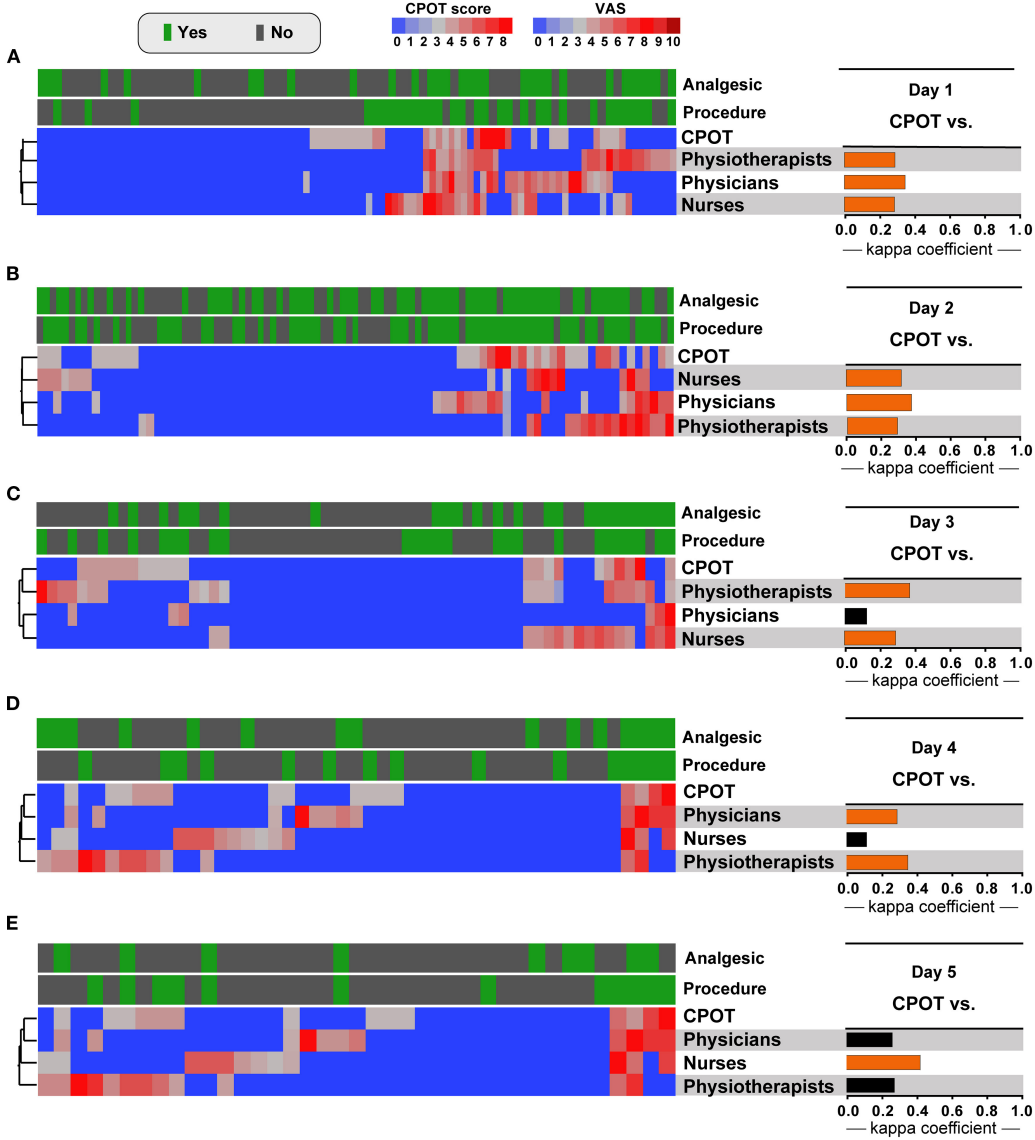
Pain assessment profile by nurses, physicians and physiotherapists and results of CPOT score in ICU patients stratified by analgesics treatment and medical procedures. The kappa coefficient of agreement between the CPOT score and pain assessment by nurses, physicians, and physiotherapists on day 1 **(A)**, day 2 **(B)**, day 3 **(C)**, day 4 **(D)**, and day 5 **(E)**. Statistically significant agreements are highlighted in orange. CPOT, Critical care pain observation tool; VAS, Visual analog scale for pain.

There was a slight agreement among professionals in the first 2 days of evaluations (Day 1 Kappa index = 0.2, 95% CI: 0.14–0.26, *p* < 0.001; Day 2 Kappa index = 0.12, 95% CI: 0.06–0.19, *p* < 0.001; Figure 2). Moreover, there was a predominance of concordance in the evaluation of the absence of pain, but a wide variability in the evaluations when the pain is present.

## Discussion

The ICU may represent a threatening environment, with unfamiliar monitoring equipment, loss of day-night cycle orientation and invasive, often painful, procedures that are now recognized as contributing factors to the development of post-ICU syndrome in survivors (9, 10). Several studies address the importance of adequate recognition of pain, as it is critical to provide appropriate sedation and analgesia to mitigate psychological and physical complications. Pain may lead to deleterious physiologic effects including sympathetic hyperactivity with subsequent increased heart rate and myocardium oxygen consumption, increased respiratory rate and hypoxemia, altered gastrointestinal motility and urinary tract function, changes in blood viscosity, coagulation and platelet aggregation, as well as decreased immune function and wound healing (11). In contrast, excessive sedation may increase the risk of venous thrombosis, prolonged mechanical ventilation and hospital-acquired infections, decreased bowel mobility, hypotension, and reduced tissue oxygen extraction capacity, leading to prolonged ICU length of stay (12, 13). Despite having a broad understanding of the consequences of inadequate pain control, the PAINT study demonstrated that pain recording appears to be of low priority by doctors (7). Our study reinforces the multidisciplinary approach to the theme, considering that nurses and physiotherapists have their methods of pain relief, in addition to pharmacological therapy, and the joint action of the health team provides complete and optimized care (14).

In Europe, a multi-center study that sought to characterize the severity and determinants of pain, the most painful procedure was the removal of a chest tube, occurring at a rate of 6.1% of the total procedures, similar to that registered in this study (1). In a prospective cohort of 1,318 patients in 44 French ICUs, 90% of patients used opioids and sedatives on the first day of hospitalization, a much higher prevalence than that found in our study (15).

Both the CPOT and the professionals' evaluations showed a low average level of pain. Despite this, only a weakly positive correlation was found between subjective pain assessments and CPOT, highlighting that, even with minimal pain, members of a multidisciplinary team performed poorly in individual pain assessments. Surprisingly, although nurses had much more contact with patients at the bedside, this did not translate into better agreement with CPOT in pain assessments. These findings contrast with the original CPOT study that found a positive, moderate correlation between self-report of pain intensity and CPOT scores, while patients underwent an experimental pain procedure, suggesting that the higher the level of pain, the higher the CPOT score. Furthermore, a validation study of CPOT demonstrated high sensitivity (86.1%) of the CPOT scale in pain detection. The poor correlation and agreement between CPOT pain assessments and the multidisciplinary team found in our study suggest that subjective pain assessment performed by health professionals is inadequate to identify the existence of pain in ICU patients, especially in those unable to report their pain. The results of our study reinforce recommendations that a validated behavioral scale should be used as an alternative to detect pain and evaluate its intensity (16–18).

Our study found that physicians, nurses, and physiotherapists pain assessments demonstrated slightly positive agreement on days 1 and 2. In addition, subjective pain assessments by health professionals were in most agreement when no pain was noted, whereas pain assessments >0 demonstrated wide variability for the same patient at the same time. The lack of at least a moderate agreement in subjective pain evaluation by physicians, nurses, and respiratory therapists suggests the absence of a common standard in the assessment of pain performed by these professionals. Therefore, the establishment of a systematic pain assessment protocol throughout the stay in the ICU may have a beneficial effect, reducing the consequences of inadequate pain management.

Our study has several limitations including a small sample in a single center and the non-individualization of pain assessments by professionals. This is balanced with the ability to evaluate multiple members of a multidisciplinary team during the study period in a more granular way with the results that complement previous studies. A few patients in our study underwent mechanical ventilation combined with rare use of sedatives, leading to limited comparisons between subjective assessments and CPOT in sedated and intubated critically ill patients. While this patient population differs from prior studies, it nonetheless demonstrated similar limitations of subjective pain assessments when compared to CPOT, even in non-intubated and minimally sedated patient populations. The gold standard for assessing the presence of unacceptable levels of pain is still self-reported pain by the patient. The CPOT was developed from retrospective chart reviews and physiological parameters were excluded in the initial assessment. In addition, the application of the tool in specific situations, such as patients with delirium, requires further evidence (19).

## Conclusion

This study identified no significant positive agreement between CPOT and bedside pain assessments by physicians, nurses and physiotherapists. The lack of agreement between interprofessional assessments in the presence of pain highlights the urgent need to properly address pain systematically in critically ill patients.

## Data availability statement

The raw data supporting the conclusions of this article will be made available by the authors, without undue reservation.

## Ethics statement

The studies involving human participants were reviewed and approved by Research Ethics Committee of the Federal University of Bahia under the number 2.249.687 and CAAE 73835317.5.0000.5577. The patients/participants provided their written informed consent to participate in this study.

## Author contributions

KA, NF, and BA were responsible for study design, implementation, manuscript preparation, and had full access to all the data in the study and take responsibility for the integrity of the data and the accuracy of the data analysis. RS, RM, IF, TC, VS, MO, AG, and SA contributed substantially to the study design, data analysis and interpretation, and writing and review of the manuscript. KA and MA were responsible for advanced statistical analysis, figure generation, and manuscript review and preparation. All authors contributed to the article and approved the submitted version.

## Funding

This study was supported in part by the intramural research program of FIOCRUZ. The work of BA was supported by a grant from NIH. IF was supported by UNEB: PICIN/UNEB. KF received a fellowship from the Programa Nacional de Pós-Doutorado, The work of BA was supported by grants from NIH (U01AI115940 and U01AI069923). BA was a senior scientist from the Conselho Nacional de Desenvolvimento Científico e Tecnológico (CNPq), Brazil. TC received a fellowship from Coordenação de Aperfeiçoamento de Pessoal de Nível Superior (Finance code: 001). Fogarty International Center and National Institute of Child Health & Human Development of the National Institutes of Health under (Award Number D43 TW009763 through a research scholarship awarded to MA) and by the NIH (U01AI069923). MA received a research fellowship from the Fundação de Amparo à Pesquisa do Estado da Bahia (FAPESB), Brazil.

## Conflict of interest

The authors declare that the research was conducted in the absence of any commercial or financial relationships that could be construed as a potential conflict of interest.

## Publisher's note

All claims expressed in this article are solely those of the authors and do not necessarily represent those of their affiliated organizations, or those of the publisher, the editors and the reviewers. Any product that may be evaluated in this article, or claim that may be made by its manufacturer, is not guaranteed or endorsed by the publisher.
