# Supplementary material for: A prospective comparison between multidisciplinary healthcare providers' clinical examination and a validated pain scale
Source: Front Pain Res (Lausanne). 2022 Aug 12;3:960216. doi: 10.3389/fpain.2022.960216 (PMC9411743; doi:10.3389/fpain.2022.960216)
Supplement: Supplementary file 1 [file Table_1.DOCX]

**Supplementary figure 1.** Bland-Altman analysis between CPOT and Health team pain assessments.
